# Paracetamol Interference in Uric Acid Levels in Uremic Patients Revealed by Monitoring Spent Dialysate

**DOI:** 10.5402/2013/515292

**Published:** 2013-10-03

**Authors:** Risto Tanner, Jürgen Arund, Ivo Fridolin, Merike Luman

**Affiliations:** ^1^Department of Biomedical Engineering, Technomedicum, Tallinn University of Technology, Akadeemia tee 5, 19086 Tallinn, Estonia; ^2^National Institute of Chemical Physics and Biophysics, Akadeemia tee 23, 12618 Tallinn, Estonia; ^3^Centre of Nephrology, North-Estonian Medical Centre, J. Sütiste tee 19, 13419 Tallinn, Estonia

## Abstract

The aim of this study was to assess removal dynamics of paracetamol (PAR), as an extraordinary chromophore in spent dialysate, upon the optical monitoring of dialysis of end-stage renal disease patients with inflammation complications. Seven dialysis sessions of different patients were followed to whom PAR was used as a pain reliever or antipyretic. Spent dialysate was sampled hourly and analyzed using HPLC with MS/MS and UV detection. Quantitative calculations were made on the basis of the peak areas on the chromatograms at 280 nm for uric acid (UA) and 254 nm for PAR and its metabolites (PAR-M). Peaks of UA, PAR, PAR-glucuronide, and PAR-sulphate were identified on the basis of specific mass spectra. Removal of PAR was found to be proportional to that of uric acid if intake of the drug by patient occurred half a day before dialysis. But disturbances of the UV-absorbance curves at 280 nm were observed related to rise of UA concentration in spent dialysate when PAR was taken by patients in the course of dialysis. The mechanism of such relation remains unknown. It was concluded that possible benefits and risks of treatment of uremic patients with paracetamol-containing drugs may need to be reassessed.

## 1. Introduction

Uric acid (UA) is known as a normal final product of human metabolism of purines, essential constituents of nucleic acids, and is normally excreted by kidney with urine [1]. High concentration of UA in serum often associates with severe chronic pathologies, such as arthritis, hypertension, and so forth [2]. Many recommendations have been published for such patients listing purine-rich food products to be avoided as well as some drugs, which are known to be associated with a raise of UA in blood, but paracetamol (PAR), a worldwide used analgesic and antipyretic drug [3], is not usually included into such lists. Paracetamol (PAR) is known as a potent enhancer of the effect of many other drugs [4] and its combination with analgesic drugs as pain reliever, including treatment of acute gout-like arthritis associated with high level of UA in blood [5]. Some observations have been published in the older literature concerning elevated analytical results of UA in serum following PAR administration, but this effect was found to be caused by interference of PAR with phosphotungstate reduction method of UA analysis only and an alternative uricase method did not confirm relationship between UA concentration and PAR administration [6, 7]. Since no strong limitation is known, PAR is dosed sometimes also to patients with renal failure as an analgesic or antipyretic drug.

The online monitoring of total ultraviolet (UV) absorbance in the spent dialysate has empowered itself as a valuable tool for continuous monitoring of a single hemodialysis session with the possibility of customizing the treatment in accordance with the physiological condition of the patient [8, 9]. The wavelength of 280 nm is commonly used in optical dialysis adequacy sensors for this purpose [10] and UA appears to be the main chromophore at this wavelenth among variety of metabolites, eliminated from the blood of end-stage renal disease (ESRD) patients by dialysis [11]. The decline of the absorbance of spent dialysate at 280 nm during dialysis session reflects quite well the removal of all small water-soluble uremic toxins and PAR with its absorbance maximum near 254 nm do not seem to substantially interfere with optical monitoring of elimination of this group of solutes from the blood of patients with end-stage kidney disease [12]. But when studying dynamics of removal of PAR from the blood of these patients, surprising parallel rise of concentrations of UA together with PAR and its metabolites (PAR+M) in spent dialysate was observed in some cases after giving PAR to patients in the course of a dialysis session. The aim of this study was to evaluate in more details removal dynamics of the paracetamol (PAR), as an extraordinary chromophore in spent dialysate, upon the optical monitoring of dialysis of end-stage renal disease patients with inflammation complications.

## 2. Subjects and Methods

The study was performed after the approval of the protocol by the Tallinn Medical Research Ethics Committee at the National Institute for Health Development, Estonia. An informed consent was obtained from all participating patients. Only standard therapeutic procedures previously appointed by doctor were used with no alterations in connection with this research. In total 17 patients, receiving thrice-weekly hemodialysis, were followed during a single dialysis session each. PAR had been prescribed to 7 of the patients as an antipyretic or as pain reliever before or during the dialysis session. Five of these patients are females and 2 are males, mean age 65 ± 13 years. 10 patients, 2 female and 8 male, mean age 62.6 ± 18.6 years not receiving PAR were followed as the control group. All patients were dialysed with polysulfone membrane dialyzers (Fresenius Medical Care, Germany) by low flux dialyzers F10 HPS with an effective membrane area of 2.2 m^2^, ultrafiltration coefficient 21 mL/h∗mmHg. The dialysis machine used in the study was Fresenius 4008H (Fresenius Medical Care, Germany), duration of sessions 4 hours with one exception (3 hours) concerning the patient number 7. The dialysate flow was 500 mL/min and the blood flow varied from 245 to 350 mL/min depending on the patient but was kept stable during dialysis session. Spent dialysate was sampled hourly or more frequently and the content of main well-known uremic toxins in samples were analyzed using high performance liquid chromatography as described previously [12, 13]. Chromatograms at the wavelengths of 254 and 280 nm were monitored and quantitative calculations were made on the basis of the peak areas on the chromatograms at 280 nm for uric acid (UA) and at 254 nm for PAR and its metabolites (PAR+M). Identification of UV peaks of UA, PAR, PAR-glucuronide, and PAR-sulfate was confirmed on the basis of characteristic mass spectra [14] by means of the MicrOTOF-Q II ESI MS/MS mass spectrometer (Bruker Daltonics, Bremen, Germany) switched online into the postcolumn eluent flow through the flow splitter Model 600-PO10_06 (Analytical Science Instruments, CA, USA). Removal ratio (RR) of UA was calculated in percentage using the formula

(1) RR=100∗(1−AendAstart),

where *A* indicates peak areas of UA on the chromatograms of the first (10 min, “start”) and the last (180 or 240 min, “end”) dialysate samples of the same dialysis session. Two-sample *t*-test was used for evaluation of differences between groups assuming unequal variances (*P* < 0.05).

## 3. Results

**Figure 1:**
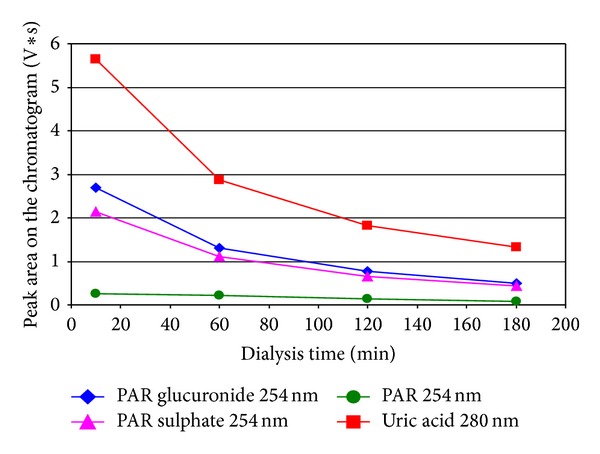
An example of change of concentrations of uric acid and paracetamol (PAR) and PAR metabolites in spent dialysate outflow during the dialysis session for the patient number 7. The patient got paracetamol thrice per day 1 g *per os*, the last dosage 10.5 hours before the dialysis.

**Figure 2:**
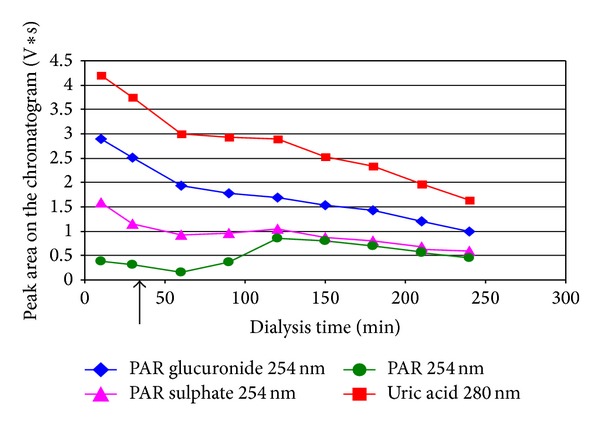
An example of change of concentrations of uric acid and paracetamol (PAR) and PAR metabolites in spent dialysate outflow during the dialysis session for the patient number 6. The patient got paracetamol thrice per day 500 mg *per os*, the first dosage on the day of dialysis 30 min after the start of dialysis session (marked by the arrow).

**Figure 3:**
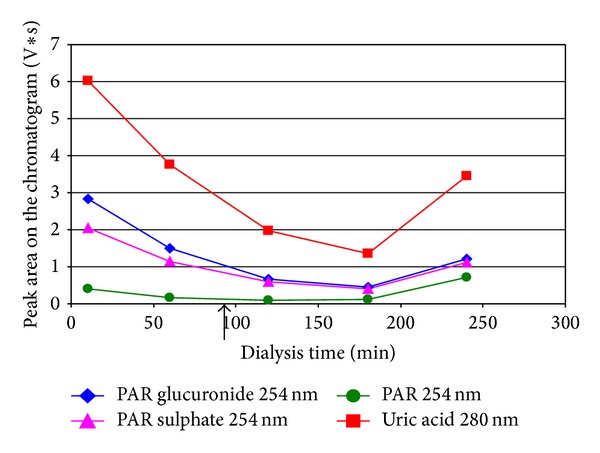
An example of change of concentrations of uric acid and paracetamol (PAR) and PAR metabolites in spent dialysate outflow during the dialysis session for the patient number 2. The patient got paracetamol thrice per day 500 mg *per os*, the first dosage on the day of dialysis 90 min after the start of dialysis session (marked by the arrow).

In the case of the patient receiving PAR overnight before dialysis, normal logarithmic-like decline of content of both UA and PAR+M during the dialysis session was observed (Figure 1). The final removal ratio (RR) of UA appeared to be the same as the average in control group (Table 1). But, unexpectedly, when PAR was given shortly before or after the start of dialysis (6 patients, Table 1), characteristic protuberance of the absorption curve of the UA elimination corresponding to the time could be seen when metabolites of PAR appeared in the spent dialysate (Figure 2). Surprisingly, a sharp increase in concentration of UA parallel to increase of PAR and metabolites in spent dialysate was observed in two cases from total six receiving PAR in the time of dialysis session (Figure 3).

The average removal ratio of UA of those 6 PAR patients 52 ± 9% (Table 1) was found on the basis of *t*-test to be significantly (*P* < 0.05) lower than the corresponding rate 78 ± 14 of patients not receiving the PAR. The initial concentrations of UA in the spent dialysate appeared to be significantly higher (with the single exception of the patient number 4) in comparison of the corresponding values in the control group (Table 1).

## 4. Discussion

The typical UV monitoring curve of elimination of small water-soluble uremic toxins by dialysis has been presented, and details of liquid chromatographic analysis of spent dialysate discussed elsewhere by our group in connection with assessment of online dialysis dose monitoring by UV absorbance [10, 12, 13]. The peak of UA is well separated from peaks of PAR and its metabolites on the chromatograms [12] and any possibility of analytical interference of PAR+ metabolites in HPLC estimation of UA content in spent dialysate seems not to be possible. Consequently, the significant rise of content of UA in spent dialysate after dosage of PAR to ESRD patients in the course of dialysis session seems to really take place at least in some cases of treatments. The chemical mechanism of such relation between PAR and UA in uremic patients remains to be clarified as well as possible good or harm to uremic patients. Uremic patients are under regular medical supervision and rarity of occasions where treatment with PAR has been indicated significantly restricting such kind of research on patients *in vivo*.

UA is not only known as a useless waste product of organism but also has proven to be involved in essential reduction and oxidation reactions in long history of human being development [15]. While UA seems to be an antioxidant in extracellular environment [16] possibly inhibiting the free radical rout of formation of AGEs [17], the UA has adverse prooxidant effect in adipocytes possibly by stimulating NADPH oxidase [18]. However, the harmful effects of high concentrations of UA in blood seems to be strongly prevalent [1], including risk for kidney diseases [19, 20]. Concerning inconsistent activities of UA, Mohandas and Johnson have concluded that “although the concept that uric acid might have a role in kidney disease once suffered a requiem, it has undergone a revival and seems deserving of additional study” [19]. The same can be concluded from our observation concerning treatment of uraemic patients with PAR: this practice “seems deserving of additional study” if PAR may significantly increase UA concentration in blood and cause additional health risk to uremic patients. An oxidative way of UA degradation concerning myeloperoxidase and hydrogen peroxide has been described in the case of cardiovascular disease [21] and PAR has been found to inhibit this enzymatic system [16]. Does it point on a possible way of direct involvement of PAR in the increase of concentration of UA in uraemic patients? Our observation of parallelism in dynamics of PAR+metabolites and UA in the spent dialysate after giving of PAR to patients with renal failure seems to support such interpretation. Alternatively, we do not know much about influence of PAR upon the distribution of UA between blood, interstitial fluid, and tissue cells. If increase of UA in dialysate after PAR treatment reflects quicker movement of UA from tissues to blood, the drug can be considered as suggested reliever of complaints of hyperuriceamic and dialysis patients.

## 5. Conclusion

Removal of paracetamol is proportional to that of uric acid and dose not interfere with on-line UV monitoring of removal of small water-soluble uremic toxins at 280 nm, if intake of the drug by patient occurs half a day before dialysis or more. But significant rise of uric acid concentration in the spent dialysate as well as disturbance of the UV-absorbance curve may be caused by intake of paracetamol during the dialysis session in spite of the great difference of absorbance maxima of these solutes. The mechanism of the relation between paracetamol and uric acid in spent dialysate remains unknown. Possible benefits and risks of treatment of uraemic patients with paracetamol-containing drugs may need to be reassessed.

## Figures and Tables

**Table 1:** Removal ratio (RR) of uric acid (UA) depending on taking of paracetamol (PAR) in the course of dialysis session by patients with end stage kidney disease.

Patient no.	Dosage of PAR		UA in dialysate		Patient	
	mg	Last dosage time*	mg/L in start sample	RR %	Sex	Age
No. 1	1000	−90**	22.19	66	F	65
No. 2	500	+95	18.11	43	M	47
No. 3	1000	+30	26.73	47	F	78
No. 4	1000	+45	7.93	47	F	76
No. 5	1000	+10	15.32	49	M	76
No. 6	500	+30	12.61	60	F	47
		Mean ± SD	17.15 ± 6.74	52 ± 9		
No. 7	1000	−630***	16.96	77	F	70
Control group	No PAR	Mean ± SD	8.87 ± 2.11	78 ± 14	8 M, 2 F, mean age 62.6 ± 18.6, 10 dialysis sessions in total	

Notes: *Time in minutes before (−) of after (+) the start of dialysis.

**Intravenous dropping 10 mg/min, 1 g total beginning from 90 min before dialysis.

***The last dosage overnight before the dialysis.

## References

[1] Álvarez-Lario B, Macarrón-vicente J (2011). Is there anything good in uric acid?. *QJM*.

[2] Merriman TR (2011). Editorial. *Current Rheumatology Reviews*.

[3] Zhao L, Pickering G (2011). Paracetamol metabolism and related genetic differences. *Drug Metabolism Reviews*.

[4] Li Wan Po A, Zhang WY (1997). Systematic overview of co-proxamol to assess analgesic effects of addition of dextropropoxyphene to paracetamol. *British Medical Journal*.

[5] Man CY, Cheung ITF, Cameron PA, Rainer TH (2007). Comparison of oral prednisolone/paracetamol and oral indomethacin/paracetamol combination therapy in the treatment of acute goutlike arthritis: a double-blind, randomized, controlled trial. *Annals of Emergency Medicine*.

[6] Wilding P, Heath DA (1975). Effect of paracetamol on uric acid determination. *Annals of Clinical Biochemistry*.

[7] Smith M, Payne RB (1979). Re-examination of effect of paracetamol on serum uric acid measured by phosphotungstic acid reduction. *Annals of Clinical Biochemistry*.

[8] Luman M, Jerotskaja J, Lauri K, Fridolin I (2009). Dialysis dose and nutrition assessment by optical on-line dialysis adequacy monitor. *Clinical Nephrology*.

[9] Castellarnau A, Werner M, Günthner R, Jakob M (2010). Real-time Kt/V determination by ultraviolet absorbance in spent dialysate: technique validation. *Kidney International*.

[10] Lauri K, Arund J, Holmar J, Tanner R, Luman M, Fridolin I, Carpi A, Donadio C, Tramonti G (2011). Optical dialysis adequacy monitoring: small uremic toxins and contribution to UV-absorbance studied by HPLC. *Progress in Hemodialysis—From Emergent Biotechnology to Clinical Practice*.

[11] Jerotskaja J, Uhlin F, Fridolin I, Lauri K, Luman M, Fernström A (2010). Optical online monitoring of uric acid removal during dialysis. *Blood Purification*.

[12] Arund J, Tanner R, Uhlin F, Fridolin I (2012). Do only small uremic toxins—chromophores contribute to the online dialysis dose monitoring by UV absorbance. *Toxins*.

[13] Lauri K, Tanner R, Jerotskaja J, Luman M, Fridolin I (2010). HPLC study of uremic fluids related to optical dialysis adequacy monitoring. *International Journal of Artificial Organs*.

[14] Hewavitharana AK, Lee S, Dawson PA, Markovich D, Shaw PN (2008). Development of an HPLC-MS/MS method for the selective determination of paracetamol metabolites in mouse urine. *Analytical Biochemistry*.

[15] Johnson RJ, Andrews P, Benner SA, Oliver W (2010). Theodore E. Woodward award. The evolution of obesity: insights from the mid-Miocene. *Transactions of the American Clinical and Climatological Association*.

[16] Koelsch M, Mallak R, Graham GG (2010). Acetaminophen (paracetamol) inhibits myeloperoxidase-catalyzed oxidant production and biological damage at therapeutically achievable concentrations. *Biochemical Pharmacology*.

[17] Wickens DG, Norden AG, Lunec J, Dormandy TL (1983). Fluorescence changes in human gamma-globulin induced by free-radical activity. *Biochimica et Biophysica Acta*.

[18] Sautin YY, Nakagawa T, Zharikov S, Johnson RJ (2007). Adverse effects of the classic antioxidant uric acid in adipocytes: NADPH oxidase-mediated oxidative/nitrosative stress. *American Journal of Physiology*.

[19] Mohandas R, Johnson RJ (2008). Uric acid levels increase risk for new-onset kidney disease. *Journal of the American Society of Nephrology*.

[20] Obermayr RP, Temml C, Gutjahr G, Knechtelsdorfer M, Oberbauer R, Klauser-Braun R (2008). Elevated uric acid increases the risk for kidney disease. *Journal of the American Society of Nephrology*.

[21] Meotti FC, Jameson GNL, Turner R (2011). Urate as a physiological substrate for myeloperoxidase: implications for hyperuricemia and inflammation. *The Journal of Biological Chemistry*.

